# Australia's insurance crisis and the inequitable treatment of self-employed midwives

**DOI:** 10.1186/1743-8462-5-6

**Published:** 2008-05-29

**Authors:** Milena Canil

**Affiliations:** 1 School of Public Health, La Trobe University, Victoria, 3086, Australia and Victorian Government Department of Human Services, 50 Lonsdale Street, Melbourne, 3000, Australia

## Abstract

Based upon a review of articles published in Australia's major newspapers over the period January 2001 to December 2005, a case study approach has been used to investigate why, when compared with other small business operators, including medical specialists, Australian governments have appeared reluctant to protect the economic viability of the businesses of self-employed midwives. Theories of agenda setting and structuralism have been used to explore that inequity. What has emerged is a picture of the complex of factors that may have operated, and may be continuing to operate, to shape the policy agenda and thus prevent solutions to the insurance problems of self-employed midwives being found.

## Introduction

During the period 2000 to 2003, Australia's major daily newspapers increasingly carried reports about a 'crisis' in insurance and the impact that upheavals in the insurance market was having on a number of professions, businesses and community groups. In terms of the health sector, the effect that insurance premium increases at the time had on the Australian medical profession, and the various policy solutions devised to address those effects, was comprehensively reported in both the Australian media and academic literature. Relatively less attention was paid to the insurance problems experienced by other health professionals. Of that group, self-employed midwives warrant special attention. Self-employed midwives are the only health professionals still unable to secure professional indemnity insurance within the private sector insurance market, and this has resulted in the loss of small businesses.

The purpose of this article is to investigate why, when compared with other small business operators, including medical specialists, governments have been reluctant to protect the economic viability of the businesses of self-employed midwives when those businesses were threatened by upheavals in the insurance market. A case study about self-employed midwives' loss of professional indemnity insurance cover, and their endeavours to regain that cover and reinstate and protect their independent status, provides the platform for this investigation. The case study will reveal that the withdrawal of professional indemnity cover may have been justified on the basis of prudent commercial decisions. However, an analysis of the case study from the theoretical perspectives of medical dominance and structural interests suggests that the reluctance of governments to assist self-employed midwives has been underpinned by the medical profession's entrenched "monopoly over the provision of obstetric services" [[1] p.143]. Drawing upon theories of agenda setting, this article will argue that political interests act to support the medical profession's dominance in this field of health care and maintain the *status quo*. Those interests have sought, either actively or passively, to suppress or eliminate any competition to the medical profession that may be posed by self-employed midwives. This article contends that the withdrawal of professional indemnity insurance cover for self-employed midwives has been a welcomed ally for the medical profession. Further, it suggests that the lack of availability of this type of insurance cover from the private market signals that the risk posed by the provision of birthing services by midwives acting independently is considered too high, a view that reinforces medicine's subordination of midwifery.

## Methods

This study uses a mixed-method approach for the collection, management and coding of data drawn from a review of news items published in Australia's major newspapers over the period January 2001 to December 2005 inclusive. The newspapers searched comprised Australia's two daily national newspapers and each of the major daily newspapers circulated within each state and territory. Newspapers circulated weekly such as the Victorian *Sunday Age *and the New South Wales *Sunday Telegraph *were also reviewed (see Table 1). Excluded from the review were regional newspapers and local community (suburban) newspapers that are circulated on a weekly basis.

**Table 1:** Major Australian newspapers

**Title**	**Frequency and Circulation**	***Factiva *first available issue**
*Adelaide Advertiser*	Daily – Adelaide/South Australia	12 January 1998
*The Age*	Daily – Melbourne/Victoria	19 January 1991
*The Australian*	Daily – National	8 July 1996
*Australian Financial Review*	Daily – National	5 April 1982
*Canberra Times*	Daily – Canberra	3 September 1996
*Courier Mail*	Daily – Brisbane/Queensland	20 January 1998
*Centralian Advocate*	Bi-weekly – Darwin, Alice Springs/Northern Territory	7 August 2001
*Daily Telegraph*	Daily – Sydney/New South Wales	8 July 1996
*Herald-Sun*	Daily – Melbourne/Victoria	23 July 1997
*Hobart Mercury*	Daily – Hobart/Tasmania	1 April 1999
*Northern Territory News*	Daily – Darwin, Alice Springs/Northern Territory	6 September 2000
*Sunday Age*	Weekly – Melbourne/Victoria	27 January 1991
*Sunday Mail*	Weekly – Adelaide/South Australia	18 January 1998
*The Sunday Mail*	Weekly – Brisbane/Queensland	21 May 2000
*Sunday Tasmanian*	Weekly – Hobart/Tasmania	21 May 2000
*Sunday Telegraph*	Weekly – Sydney/New South Wales	4 August 1996
*Sunday Territorian*	Weekly – Darwin, Alice Springs/Northern Territory	6 September 2000
*Sunday Times*	Weekly – Perth/Western Australia	12 August 2001
*Sydney Morning Herald*	Daily – Sydney/New South Wales	1 September 1986
*West Australian*	Daily – Perth/Western Australia	2 August 1996

Using the terms *midwives and indemnity *and *midwives and insurance*, the online tool *Factiva *was used to conduct the search of the print media as it provides access to full text articles. A total of 244 items of relevance to the study comprising 200 news and feature articles, 35 letters to the editor and 9 editorial and opinion pieces were downloaded, printed and filed chronologically in preparation for data coding.

Using the grounded theory techniques developed by Strauss and Corbin [2], the data was indexed according to key themes with deeper coding drawing out a series of sub-themes. These themes and any notations made onto the documents were then compiled into a case record [3]. From the case record a decision was then made in relation to whether additional information was needed from other sources, such as government department websites, or by the generation of additional search results using *Factiva *so that an in-depth case study could be written [4].

## Background

A health care provider owes a patient a duty of care because that patient is reliant upon the professional skills of that provider. If that duty of care is breached and an injury to the patient arises, then, under the system of tort law that operates in Australia, that patient may attempt to financially recover from that health care provider damages that have resulted because of that breach. Having determined that a breach of that duty of care occurred and, as a result the patient was injured, the concern for the law of torts is then to determine how to best allocate the losses [5].

In modern Australian society, insurance operates to stop the mere redirection of loss from the injured to the one found to have caused the injury. Instead, insurance distributes the loss "...among all policy holders carrying insurance on [that particular] type of risk" [[5] p.10]. The principle underpinning such 'loss distribution' is that "if a certain type of loss is looked upon as the more or less inevitable by-product of a desirable but dangerous activity, it may well be just to distribute its costs among all who benefit from that activity..." [[5] p.9]. Consequently, while the providers of the activity will initially bear the cost of losses through their insurance premiums, ultimately these costs will be distributed to other beneficiaries of that activity, that is, consumers, who are likely to be charged higher prices for those services. The market can absorb this transfer of premium costs to a point. However, once that point is reached, continuation of that activity will no longer be sustainable.

Professional indemnity insurance, like other insurance products, provides a mechanism for loss distribution. In the event that an injury arises from a breach of a health care provider's duty of care for a patient and a claim for damages against that provider is found, then that provider's professional indemnity insurance will protect his or her assets while at the same time provide compensation for the injured. The desire to protect the interests of both the midwife and the patient underpins the laws enacted in some Australian jurisdictions requiring midwives to hold professional indemnity insurance cover as a condition of professional registration.

For the majority of Australian midwives, satisfying such a condition of registration was, and has continued to be, relatively unproblematic, as their professional indemnity insurance has been arranged through their public or private hospital employers. For those midwives employed to assist obstetricians or general practitioners in their practices, those medical practitioners were, and continue to be, able to arrange insurance cover for 'practice staff' through their medical indemnity insurer. A similar situation had, until 2002, existed for nursing agency-employed midwives where the agency was able to secure insurance cover on their behalf.

In the minority were those midwives who conducted their own practices; those midwives are known as independent or self-employed midwives. For them, satisfying any conditions imposed on their registration requiring that they hold adequate professional indemnity insurance cover became somewhat problematic, if not impossible. Up until mid-2001, some 80 of Australia's 200 self-employed midwives purchased their professional indemnity insurance cover from Guild Insurance through an arrangement with the Australian College of Midwives Incorporated (ACMI) [6]. The option to purchase professional indemnity insurance cover through this arrangement was available for all midwives who were members of ACMI. The premiums charged by Guild Insurance ranged from approximately $1,000 to $1,500 *per annum *[7].

With the subsequent withdrawal from the market of their principal insurer, and the increasingly unaffordable levels of professional indemnity premiums offered by those remaining insurers willing to extend such cover to this group of health professionals, the problem for self-employed midwives became dire. Subsequently, within a short space of time, professional indemnity cover for those midwives could no longer be purchased from any insurer either in Australia or elsewhere in the world. Faced with the prospect of continuing to operate their businesses uninsured in terms of their professional indemnity risk, the majority of Australia's self-employed midwives eventually stopped providing services.

## Case study

### A 'crisis' in insurance

During the period 2000 to 2003, as the cost of public liability insurance premiums increased, so too did the frequency of media reports about the viability of community groups and small to medium sized businesses. Their futures were threatened because of their inability to either meet premium increases which, in some cases, amounted to a doubling in price within a 12 month period, or because the scope of their insurance cover was reduced or their existing cover was withdrawn. Providers of health care services, in particular medical practitioners, experienced similar effects as the cost of medical indemnity insurance (a type of professional indemnity insurance) premiums escalated over the same period.

As the media portrayed a 'worsening insurance crisis', businesses, including private sector health care providers, consumers, lawyers, and insurers, blamed one another for Australia's public liability and professional indemnity insurance woes. Governments were increasingly called upon to find solutions that would stem the rise in premiums and strengthen the prospects for longevity of the Australian insurance market by attracting more insurers into its fold. National forums on insurance issues were held, reports commissioned and vigorous debates waged about the reasons behind the crippling premium increases and possible solutions. In the meantime, many in the medical profession threatened or took action to withdraw their services and some retired prematurely. Businesses such as tourist railways, horse trail riding and adventure sports closed, and events such as community festivals and school fetes were increasingly cancelled.

While debates about causes and solutions continued, Australia's state and territory governments tried to persuade insurance companies to open their books for examination [8] and demanded greater Australian Government regulatory scrutiny of the insurance industry [9]. Insurers responded, calling for nationally consistent reforms to the laws of negligence that would curtail people's ability to sue for personal injuries thereby leading to a reduction in the number of claims made, and impose limits on the level of damages awarded by courts thereby reducing the amounts that they would have to pay out in compensation [10].

As an unprecedented state/territory-based reformation of Australia's tort laws was set in motion, driven by social and economic imperatives state and territory governments took more immediate steps to protect businesses and to preserve community cohesion and social activities. They brokered or instituted a variety of schemes designed either to provide public liability cover at affordable prices, or provide funding assistance for community organisations and a variety of businesses to purchase insurance cover. For many businesses and community organisations these initiatives were their lifelines. However, economic imperatives appeared insufficiently strong to compel state and territory governments to assist all types of small businesses. State and territory governments were reluctant to assist self-employed midwives, also small business operators, faced with insurance problems. Further, calls for help to the Australian Government by ACMI and various midwifery and consumer groups appeared to be ignored. Unlike the series of actions taken by the Australian Government during this same period to support medical practitioners and their insurers in terms of medical indemnity insurance arrangements, the Australian Government seemed unwilling to accept the insurance plight of midwives as an issue worthy of its intervention.

### Withdrawal of insurance cover for midwives

When Guild Insurance advised ACMI and its members in June 2001 that professional indemnity insurance cover for self-employed midwives would no longer be available after 1 July 2001, it explained that its decision to withdraw from the midwifery insurance market was based upon a review of its portfolio and a re-evaluation of the risks of midwifery. Guild Insurance advised that it was making a "responsible decision" and that such cover could be better provided by others in the market [11]. A significant consideration for Guild Insurance was that even though it had not received any claims against an insured midwife, the number of self-employed midwives that it insured was small and the premiums collected were insufficient to cover even a single large damages award [12].

By September 2001, St Paul Insurance joined Guild Insurance in withdrawing cover for midwives. St Paul Insurance was the underwriter for the Victorian branch of the Australian Nursing Federation (ANF), which provided professional indemnity cover for its members. Like Guild Insurance, St Paul Insurance advised that it regarded the cost and risk of potential claims as too great (despite no claims having ever been made against a Victorian midwife in the 10 years that the ANF had provided cover) [13]. Some seven months after St Paul Insurance withdrew its cover for Victorian midwives, the Tasmanian branch of the ANF advised its members that professional indemnity cover had been withdrawn for self-employed midwives [14]. ACMI and the Victoria and Tasmanian branches of the ANF approached other insurers. Each insurer, in turn, either declined to insure midwives or offered unaffordable premiums [13,14].

For the majority of Australia's self-employed midwives, the loss of professional indemnity insurance cover as the term of their current policies ended meant the loss of livelihood. With no insurer to write new policies, self-employed midwives had to decide whether or not to practice uninsured. Those who made the choice to cease their practices then had to face the difficult task of advising their pregnant clients to make alternative arrangements for their ongoing care and birth of their children. Midwives who had operated their own businesses for years were forced to retire, pursue an alternative career or find work in a hospital. Various media reports [15] suggested that some midwives decided that they would continue to practice even though they were not insured. This would potentially expose their personal assets to risk (unless they had divested themselves of those assets) [16] and, in some jurisdictions, practicing without insurance cover could result in deregistration [17].

Through various newspapers, existing or prospective clients of self-employed midwives expressed their outrage over the decisions of the insurance industry. Many reported that they were being denied access to the continuous care of a midwife of their choice or that they were being 'forced' into giving birth in a hospital under the care of an obstetrician. In terms of the latter, this was an unpalatable option given their belief that birthing is not a medical event and that birthing in a hospital equated with intervention. Many believed that Australian governments were standing by and letting insurance companies dictate how and where women birth. Some, however, refused to relinquish what they believed to be their basic human right to choose to birth in the 'safety' of their own home and revealed that they were determined to give birth at home without the support of a midwife [18-20].

With hopes fading of finding an Australian insurer prepared to underwrite the liabilities of self-employed midwives, ACMI and the ANF were forced to look overseas. However, it quickly became apparent that the search would be fruitless. ACMI and other midwifery and consumer groups turned to Australia's state, territory and federal governments in a bid to find an insurance solution for Australia's self-employed midwives.

### Demands for government action

The initial demands made on Australia's governments by midwives and consumers were essentially three fold:

(i) provide professional indemnity cover for self-employed midwives;

(ii) expand public sector maternity services to include home births thereby giving women an alternative to birthing in a hospital setting; and

(iii) introduce a national, no fault compensation scheme for those injured through the negligence of others [18,21,22].

Demands that jurisdictions provide publicly funded home births or step in as insurers of last resort generated mixed reactions. The idea of a no fault scheme, which potentially could address the plight of self-employed midwives in the longer term, appeared to fail to ignite the imagination of governments.

By March 2002, difficulties in securing professional indemnity cover for midwives had extended to universities. For instance, the withdrawal of insurance coverage for midwifery students of Flinders University in South Australia left students unable to complete the clinical component of their studies [23]. Nursing agencies faced a similar predicament. Unable to secure professional indemnity cover for their midwives, nursing agencies were only able to offer midwives general nursing work [24]. Eventually some agencies were able to secure cover through a Lloyds of London underwriter. However, in at least one case, the cost of the premium was inflated to at least five times that of the agency's original policy [25].

### Escalation of the medical indemnity 'crisis'

As insurers were withdrawing from writing professional indemnity cover for midwives, the so called 'medical indemnity crisis' affecting the Australian medical profession was escalating. Responding to increasing calls for government action, the Prime Minister announced a national forum to discuss medical indemnity issues and possible solutions. The forum, which was chaired by the then Australian Government's Minister for Health & Ageing, Senator Kay Patterson, was held in Canberra on 23 April 2002. The forum brought together federal, state and territory health ministers, and representatives of the medical colleges, the Australian Medical Association, medical defence organisations and a range of other key stakeholders.

Following the national forum on medical indemnity, the Australian Government implemented a series of initiatives designed to support medical practitioners and their insurers (for a comprehensive account of the 'medical indemnity crisis', the effect of the broader 'insurance crisis' and governments' responses see [26,27]). The Australian Government provided substantial financial support to Australia's largest medical defence organisation, United Medical Protection, which entered into provisional voluntary liquidation in April 2002 [26]. Additionally, in the wake of the 2001 collapse of HIH Insurance Ltd., the Australian Government introduced a series of legislative changes designed to strengthen the prudential regulation of the insurance industry. Those changes included requiring medical defence organisations (which had operated as doctor-owned funds that would pay claims made against a member doctor at the organisation's discretion [27]) to operate as insurers and thereby become subject to the scrutiny of the Australian Prudential Regulation Authority [28]. The Australian Government also established a 'Panel of Eminent Persons' to conduct a 'Principles based Review of the Law of Negligence'. The Panel was principally charged with examining "a method for the reform of the common law with the objective of limiting liability and quantum of damages arising from personal injury and death" [[29] p. viii]. Although its remit was broad, in terms of health professionals the review panel's focus was on medical practitioners.

By October 2002, a number of steps were also taken to address rising premiums and provide the medical profession with more certainty regarding insurance. Direct financial assistance was provided to medical practitioners and their insurers through a series of initiatives announced by the Prime Minister which, over the subsequent twelve months, would be further refined. Those initiatives and their refinements included:

▪ the Premium Support Scheme which would assist eligible practitioners by making payments on their behalf to medical indemnity insurers thereby resulting in reduced premiums being charged to those practitioners [30];

▪ the High Cost Claims Scheme designed to reimburse medical indemnity insurers for half of the amount paid out on a claim above $300,000 up to the limit of the practitioner's cover [31]; and

▪ the Exceptional Claims Scheme which would meet the full cost of any claim that exceeded the level for which a practitioner was insured [32].

### Launch of the National Maternity Action Plan

While the Australian Government was rolling out a series of initiatives that would cost taxpayers in excess of $600 million over four years and that were fundamentally designed to dissuade doctors from carrying out their threats of resignation [33], midwives and consumers reinvigorated their lobbying of governments. On 24 September 2002, the *National Maternity Action Plan for the Introduction of Community Midwifery Services in Urban & Regional Australia *(the Plan) was launched [34].

Developed by midwives and consumers, the Plan was designed to influence changes to the way in which maternity services were delivered across Australia by providing governments with an implementation strategy. The Plan's authors advised that although a series of government-led reviews into maternity services since the mid-1980s had recognised the benefits of midwifery led care, those reviews had failed to shift the medical model of care (where the general practitioner or obstetrician acts as the lead professional) as the principal way in which maternity services were being delivered in Australia [[34] p. 11]. Drawing upon international research and practice, the Maternity Coalition, the Australian Society of Independent Midwives and Community Midwifery WA Inc [[34] p. 6] argued that "midwifery led care is the most appropriate care for the majority of pregnant women", and that "maternity services should be reformed to provide universal access to continuous care by community midwives through the public health system".

Included in the Plan's recommendations for governments was one that called on the Australian Government to

introduce a Policy on Maternity Service Provision and an Implementation Framework that addresses structural reforms such as funding, legislation, standards of care and indemnification to enable planned and sustainable implementation of community midwifery programs in both urban and regional areas as a matter of priority [[34] recommendation 2, p. 5].

Other recommendations called on the Australian Government to review the Medicare Schedule to "include midwives as legitimate experts in the provision of maternity care for women" [[34] p. 5] and for all Australian governments to "implement the necessary legislative changes to enable midwives to order tests and prescribe drug therapy already commonly used in pregnancy, labour and birth" [[34] p. 5].

## Results

The case study has revealed that the substantial financial support directed at United Medical Protection, the various initiatives designed to reassure and support medical practitioners and their insurers, the strengthening of the prudential regulation of the insurance industry, and the changes to Australia's tort laws were among the series of reforms designed to stabilise the cost dynamics of the insurance market in Australia and make it attractive to overseas insurers. This section will give further consideration to the importance of the insurance industry to the Australian economy in an attempt to explore whether this provided any guarantee that governments would assist self-employed midwives in securing and retaining professional indemnity insurance cover. It will be shown that while the Australian Government avoided being drawn towards addressing self-employed midwives' insurance issues, Australia's state and territory governments reluctantly intervened – often only because it suited their own interests to do so. In keeping with the notion that a stable insurance industry and the availability of insurance is important to economic activity, this section will also explore the impact that the loss of professional indemnity insurance cover for self-employed midwives had on those small businesses.

### Was the importance of the insurance industry and insurance products to the Australian economy a guarantee that governments would extend assistance to self-employed midwives?

In terms of premium income, the Australian insurance market is the world's twelfth largest [35] and in 2003–04 comprised some 4.0 per cent of Australia's Gross Domestic Product [36]. The financial contribution of the insurance industry to the Australian economy, especially in terms of the number of people it employs, total assets, net premium revenue and after tax operating profit [37], provided significant impetus for all Australian governments to take action when financial difficulties and other stresses beset the insurance industry over the 2000 to 2003 period. Moreover, Australian governments recognised that

the true significance of the insurance industry lies in the fact that if it didn't exist a large proportion of the rest of the economy wouldn't exist either. Without a reliable mechanism for pooling and transferring risk, much economic activity simply would not take place. Neither would a lot of social activity... [[37] p. 3–4].

Consequently, Australian governments devised a series of policy solutions that, in the short term, would protect the viability of businesses that were imperilled or enable governments to intervene where the cessation of community activities had the potential to result in the disruption and loss of community cohesion. In the health sector those solutions included the Australian Government's introduction of a series of initiatives designed to assist medical practitioners secure and maintain medical indemnity insurance coverage, as well as protecting the viability of their insurers. Longer-term solutions that were devised included the Australian Government's strengthening of the prudential regulation of insurers, and the unprecedented reformation of tort laws by the states and territories.

Governments also reacted to cases of what was considered as genuine 'market failure' [37]. For instance, when the insurance market withdrew cover for terrorism risk the Australian Government recognised that the market was failing to deliver "the 'socially optimal' amount of a product or a tolerable substitute" [[37] p. 10]. It therefore established the Australian Reinsurance Pool Corporation through which "insurers [would] be able to reinsure their exposure to liability, under eligible insurance contracts, for losses arising from declared terrorist incidents" [38].

Nothing in the public domain indicates that any Australian government admitted that the insurance market's withdrawal from the provision of professional indemnity cover for self-employed midwives and the consequential loss of small businesses was a case of market failure. What is evident from the public domain is the general reluctance on the part of all Australian governments to render assistance to self-employed midwives to protect the economic viability of their businesses and their independent status. Also of note is the determination of all jurisdictions to shift responsibility for finding a solution from one level of government to another. In fact, the constitutional division of power and the complex relationship between the Australian Government and the governments of the states and territories has indeed provided a convenient avenue for such "...buck passing and lack of accountability" [[39] p. 28].

The Australian Government avoided being drawn into the search for either a short or long term solution to the self-employed midwives' professional indemnity insurance problem. From the Australian Government's perspective, there was little financial incentive for it to assist a small group of health professionals for whom it has no responsibility. Unlike members of the medical profession who are predominantly paid by the Australian Government through Medicare, Australia's national health insurance scheme, the Australian Government has no financial relationship with self-employed midwives and thus no responsibility for supporting the services that those self-employed midwives provide.

The Australian Government's disinterest in assisting self-employed midwives was illustrated by its response to the Plan [34], and its refusal to invite ACMI or any other midwifery group to participate in the medical indemnity forum held in April 2002 [40]. In relation to the Plan, the then Australian Government's Minister for Health & Ageing, Senator Kay Patterson rejected calls to extend Medicare to midwives and the then Parliamentary Secretary to the Prime Minister, Jackie Kelly, not only expressed her concerns about the Medicare proposal but stated that she had "reservations about...using government funds to help midwives caught out in the medical indemnity crisis" [41]. In addition, Senator Patterson stated that the Australian Government had no plans to increase its role in community midwifery services as that was a principal responsibility of the states and territories [42]. As for the medical indemnity forum, Senator Patterson justified the midwives' exclusion on the basis that the forum was concerned with medical indemnity rather than professional indemnity, and that she would deal with liability issues for other health care professionals in due course [43]. However, following the medical indemnity forum, the focus of the Australian Government remained squarely on the medical profession.

In response to local pressures, some state and territory governments capitulated and intervened to provide self-employed midwives with varying degrees of assistance. The West Australian Government was one of the first jurisdictions to offer some assistance to that State's self-employed midwives. In August 2001, the West Australian Health Minister announced that the Department of Health would employ those midwives engaged in the Fremantle Community Midwifery Program. The Government insurer, RiskCover, would then cover the professional indemnity of the Program's employed midwives until a private insurer could be found [7]. This interim arrangement eventually became permanent with RiskCover providing insurance directly to midwives working in the Program [[44] p. E2515].

In June 2003, the NSW Health Department (NSW Health) indicated that it was considering implementing midwifery-led care models in NSW public hospitals where midwives would be assigned to low risk patients [45]. By March 2004, this proposal had been extended to one where NSW Health (or NSW public hospitals or area health services) would employ midwives to provide publicly funded homebirths for healthy women without medical complications who choose to birth at home [46].

Similarly, the Northern Territory Government responded when its hand was eventually forced by the Northern Territory Nurses' Board's application of legislative provisions requiring midwives to hold professional indemnity insurance as a condition of registration. The Board's decision to refuse to renew the registration of self-employed midwives resulted in one of those midwives seeking legal redress of the Board's decision, and public demand for the government to intervene and provide professional indemnity cover for midwives [47]. The Northern Territory Government's response included the provision of professional indemnity insurance cover for self-employed midwives through the Department of Health & Community Services (with the provision of cover being contingent upon employment in the public sector), strengthening of the community midwifery program, the provision of training in advanced obstetrics for midwives and medical practitioners and an outreach antenatal service for remote communities [48].

Shortly following the announcement of the NSW proposal, an initiative to make midwives the primary carers for pregnant women in the public hospital system was announced in Victoria [49]. In its three-level system of maternity services, all women experiencing uncomplicated pregnancies and not requiring ongoing medical specialist supervision would have access to primary maternity services delivered through Victoria's public hospitals. Those services would provide women with one-to-one continuous care of a midwife through their pregnancy, labour and postnatally. Women requiring medical care would be provided with secondary or tertiary level services according to their level of risk, with midwives still involved in their antenatal, labour and postnatal care [50].

In spite of these local initiatives, the states and territories, in the main, were unwilling to introduce measures to reinstate the independent, small business status once available to self-employed midwives. The West Australia initiative notwithstanding, state and territory governments generally avoided taking on private sector risk. Their unwillingness to step in as insurers of last resort for self-employed midwives was justified by their belief that if they were to indemnify one practitioner group in the private health sector, the 'flood gates would open' leading other practitioner groups to seek similar coverage [51].

However, concerns about the 'opening of the flood gates' did not hamper action in the past when, for instance, the Victorian Government in mid-1996 established a "...financially viable and legally secure alternative insurance arrangement for general practitioners in rural Victoria whose medical indemnity cover has been subjected to price increases, particularly in the areas of obstetrics and anaesthetics" [52]. Likewise, there appears to have been no hesitation on the part of the states and territories to assist other small business operators and community groups that were unable to afford or secure public liability insurance. For instance, the Victorian Government provided financial assistance to adventure tourism operators for the preparation of risk management plans and audits, and helped facilitate a group insurance scheme for community organisations [53]. The West Australian, Queensland and South Australian Governments also established similar group insurance schemes for not-for-profit and community organisations [53]. In terms of finding solutions to the public liability insurance problems of these businesses and groups, state and territory governments were prepared to respond on an individual jurisdiction basis. However, the states and territories maintained that the midwives' inability to secure professional indemnity insurance cover was a national problem warranting a national, rather than a jurisdiction by jurisdiction, response [24].

An opportunity for negotiating a national response to the midwives insurance problem was, at the time, available – namely, through the Australian Health Ministers' Conference (AHMC) and Australian Health Ministers' Advisory Council (AHMAC). In fact, it was Australia's health ministers who were lobbied by ACMI in an attempt to have the midwives' insurance issue placed onto the AHMAC agenda [[44] p. E250]. However, nothing can be readily found in the public domain to suggest that the midwives' issue was considered either by AHMC, AHMAC or its Jurisdictional Working Party on Medical Indemnity or its Consultative Forum [54].

### Did the loss of professional indemnity insurance cover have any impact on home birthing trends?

In spite of the inability of self-employed midwives to secure professional indemnity insurance cover, and the Australian print media regular reporting on the cessation of service delivery by self-employed midwives with the consequential loss of small businesses, home birthing in Australia has continued. A review of perinatal statistics published annually by Australian states and territories demonstrates that while the majority of Australian women birth in hospital settings (including birth centres which are managed by midwives and are located within hospitals or within close proximity to a hospital) some choose to birth at home. It is recognised that the data on planned home births may include those homebirths attended by medical practitioners. It is also recognised that attending women birthing at home comprises only one part of the scope of services provided by self-employed midwives. However, the number of planned home births in Australia not only provides an indication of the size of the market served by self-employed midwives, but can also be used to gauge whether the withdrawal of professional indemnity insurance cover for self-employed midwives has had any discernable impact on home birthing.

**Figure 1:**
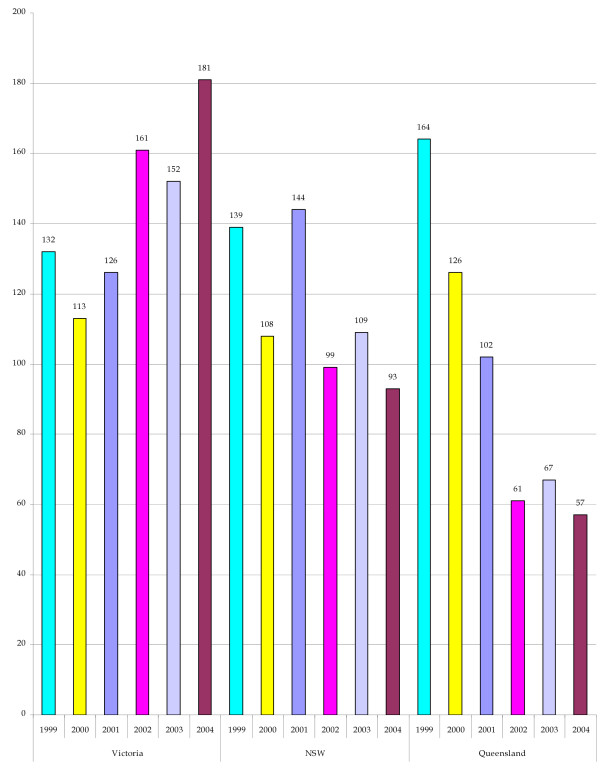
**Number of planned home births, 1999 to 2004, Victoria, NSW and Queensland**. Source: [82-92].

Figure 1 illustrates the available data on planned home births reported in Australia's most populous states of Victoria, New South Wales and Queensland. The data presented in Figure 1 and Table 2 are drawn from the years 1999 to 2004 with the period 1999 to 2000 being that immediately prior to the insurance market's withdrawal of professional indemnity insurance cover for self-employed midwives. The years 2001 to 2004 cover the period from when professional indemnity insurance cover for self-employed midwives was being withdrawn and for the years during which no cover has been available.

**Table 2:** Number of planned homebirths as a proportion of all births, 1999 to 2004, Victoria, NSW and Queensland

	**Year**	**Number of planned home births**	**Total number of births**	**Planned home births as proportion of all births**
**Victoria**	**1999**	132	61 586	0.21%
	**2000**	113	61 569	0.18%
	**2001**	126	61 064	0.21%
	**2002**	161	61 959	0.26%
	**2003**	152	62 305	0.24%
	**2004**	181	62 348	0.29%
				
**NSW**	**1999**	139	85 967	0.16%
	**2000**	108	86 460	0.13%
	**2001**	144	84 379	0.17%
	**2002**	99	84 587	0.12%
	**2003**	109	85 032	0.13%
	**2004**	93	84 288	0.11%
				
**Queensland**	**1999**	164	48 042	0.34%
	**2000**	126	48 524	0.26%
	**2001**	102	48 908	0.21%
	**2002**	61	48 324	0.13%
	**2003**	67	49 512	0.14%
	**2004**	57	50 051	0.12%

Source: [82-92]

Table 2 provides information on the reported number of planned home births as a proportion of total births for the period 1999 to 2004 for the States of Victoria, New South Wales and Queensland. The Victorian data for 1999 and 2000 indicate that the proportion of planned home births reported was 0.21 per cent and 0.18 per cent of all births in that State for those years respectively, with a decline in the actual number of planned home births in 2000 compared with 1999. New South Wales' data for the same period show that the proportion of planned home births was slightly lower than that for Victoria, with 0.16 percent of all births in that State in 1999 being planned home births. The New South Wales data show that the proportion of planned home births slightly decreased to 0.13 per cent of all births in that State for 2000. Like Victoria, the actual number of planned home births reported in New South Wales had also declined in 2000 compared with the previous year. A similar decrease in the actual number of reported planned home births is noted for Queensland for the 1999 to 2000 period. However, the Queensland data show that the actual number of reported planned home births in Queensland was higher than that reported for Victoria and New South Wales for both 1999 and 2000. The Queensland data also show that the numbers of planned home births, as a proportion of all births reported in that State for 1999 and 2000, were higher than the proportions reported for Victoria and New South Wales for the same period. However, like New South Wales in 2000, Queensland too showed a slight decrease in the number of reported planned home births as a proportion of all reported births in that year.

Based upon the assumption that self-employed midwives attended all of the planned home births in the three states in 1999 and 2000, and that the data are reasonably indicative of the number of planned home births relative to the number of all births across Australia, the self-employed midwives' market prior to the loss of professional indemnity insurance cover was small.

The withdrawal of professional indemnity insurance cover for self-employed midwives and the lack of availability of that cover for the period 2001 to 2004 had a variable impact on the number of reported planned home births in Victoria, New South Wales and Queensland. In Victoria, the number of planned home births, as a proportion of all births, remained fairly constant. However, the actual number of reported planned home births generally increased over the 2001 to 2004 period. In New South Wales, the reverse is noted with the actual number of reported planned home births over the 2001 to 2004 period showing a general decrease. Of the three States, the Queensland data show the most noticeable decline in terms of the actual number of planned home births reported for the 2001 to 2004 period and in the number of planned home births as a proportion of all reported births in that State.

In spite of the loss of professional indemnity cover for self-employed midwives, planned home birthing has continued in Victoria, New South Wales and Queensland. This could be attributed to:

(i) medical practitioners attending all of the reported planned homebirths; or

(ii) home birthing services being provided through the public hospital system and all of the deliveries being attended by hospital-employed midwives and/or medical practitioners;

(iii) self-employed midwives continuing to practice while uninsured; or

(iv) women birthing at home unassisted or with the assistance of a support person who is not a qualified midwife (either because self-employed midwives are unwilling to provide services while uninsured or those who are willing to work uninsured are unable to take on additional patients).

The most likely explanation is that these data reflect all four scenarios.

In some areas, private sector general practitioners who practice obstetrics may be assisting women who choose to birth at home. Similarly, in New South Wales, where home birth services are available through the public hospital system, hospital-employed medical practitioners and/or midwives may be attending home births. The likelihood that some women have birthed at home unassisted rather than in a hospital or birth centre cannot be rejected [55]. Equally, the determination of midwives to risk registration and continue to provide services while uninsured cannot be dismissed [56]. Arguably the data also support the likelihood that there are women in Australia prepared to accept greater responsibility for the management of their own pregnancies and childbirth, and employ midwives who carry no professional indemnity insurance cover. For these women, a confinement that offers continuity of care takes precedence over concerns for the consequences of any harm that may arise from the negligence of the midwife [56].

## Discussion

The central concern for this article has been why, when compared with other small business operators, including medical specialists, Australian governments have appeared reluctant to protect the economic viability of the businesses of self-employed midwives. A general observation about government decision making is that "decision makers are not simply forced by events, interest group pressures, or external agencies to make particular choices; generally they have a significant range of options in the management of public problems – including at times the option of not addressing them" [[57] p.2].

From the Australian Government's perspective, the lack of any economic relationship with self-employed midwives, the small size of the self-employed midwives' market, the existence of a mainstream alternative to home birthing and care by a self-employed midwife, and the responsibility of state and territories for community midwifery services provides reasonable grounds for not rendering any assistance to self-employed midwives. On the face of it, the Australian Government's lack of action to address the professional indemnity concerns of self-employed midwives – essentially adopting the 'do nothing option' – can thus be easily explained.

However, such a general observation cannot account for the varied reactions of Australia's state and territory governments. In fact, when those reactions, as well as the Australian Government's inaction, are explored from various theoretical perspectives, any notion that there may be a simple explanation to this article's question quickly gives way to a complex picture of underlying motives.

### Framework for the exploration of Australian governments' reactions

Although situated within the political decision-making processes of the United States of America, Dye [58] provides two useful approaches for exploring how problems are identified and elevated onto the policy making agenda for government decision-making: agenda setting from the bottom up and agenda setting from the top down.

Dye's 'bottom up' approach is based upon pluralist notions that, in an open society, a variety of individuals or groups outside of government can identify issues for discussion and gain the support of others, including the support of government officials, and influence decision making and the placement of their issues onto the policy agenda [58,59]. However, such views do not account for the differences in the influence wielded by various groups, all of whom are vying for space on that agenda [60], including those who may be supported by political leaders [61]. In addition, such a perspective fails to account for the activity of groups focused on preventing items reaching the agenda or 'negative blocking' so that they "preserve prerogatives and benefits that they are currently enjoying, blocking initiatives that they believe would reduce those benefits" [[62] p. 52].

In light of this, Dye's [58] alternative 'top down' approach may better reflect the complex of interests, power and influences that compete for the attention of policy makers. This approach is based upon an elitist model of agenda setting whereby particular groups decide which problems will be placed onto the policy agenda for the attention of government. These groups are situated within government and discuss problems and ideas within professional circles and with policy elites [60]. These policy elites, suggests Dye [58], are leaders in business, finance and the like who act to advance their interests or act to protect those interests against potential threats. Proponents of such a view also suggest that government decision makers and 'policy leaders' within government rely upon their subordinates situated within government bureaucracies to passively provide advice and implement decisions [63].

Given that the reactions of the jurisdictions have varied, it is important not to limit this investigation to the groups situated within government and those professional circles and policy elites with whom issues for the policy agenda are discussed. In fact, it is plausible that the subordinates who are situated within government bureaucracies may not be acting as passively as proponents of the elitist model of agenda setting suggest. Consequently, the framework that will be adopted will examine the reactions of Australian governments from a top down perspective as well as a bottom up perspective.

### How does a 'top down' perspective explain the reactions of Australian governments?

#### Governments, policy elites and the power of the medical profession

In Australia, the medical profession is included amongst the policy elites. Its position as the most powerful occupation in the health care workforce guarantees its ability to influence government policy [1,64]. That position has been achieved through a process of evolution in which the medical profession has gained professional autonomy, authority over other health professions and an ability to shape "...society's beliefs about health problems and how they should be managed" [[1] p. 48]. Willis argues that the state has supported the medical profession's rise to, and indeed maintenance of, its position in the health care system "primarily by the provision of statutory registration legislation which has ensured the subordination, limitation or exclusion of the medical professions' competitors" [[65] p. 202]. This support, has at least in part, been assured because of "class allegiances between the profession and conservative political forces" [[66] p. 287].

Control over the management of the childbirth process, a lengthy contest between medical practitioners and midwives [65,67], serves to illustrate the medical profession's achievement of its position of professional autonomy and authority over another health profession. Willis attributes medicine's ascendancy over midwifery to a process of subordination and the medicalisation of childbirth rather than to "advances in the technology associated with childbirth" [[65] p. 122]. He argues that medicine's subordination of midwifery was facilitated by midwifery's incorporation into nursing, an occupation that was already subordinate to medicine [65]. Equally he considers the medical profession's waging of 'ideological warfare' in discrediting female midwives as "ignorant and dangerous" [[65] p. 102], and medicine's historical exclusion of women from formal medical and surgical training as significant factors in the subordination process. Further, while Willis [65] argues that the predominance of women in the occupation of midwifery also facilitated its subordination by the male dominated medical profession, the 'struggle' between midwifery and medicine was not solely gender related. A 'class struggle' also occurred which saw a shift in attendance at childbirth by "working class women to attendance by middle class men" [[65] p. 93]. Willis suggests that "associated with this change in attendance [was the] transition from home to hospital as the location of childbirth" [[65] p. 93], a transition that placed childbirth in a setting that was already under medical control. Birthing in a hospital has essentially enabled the medical profession to position itself to manage the whole of the confinement [65].

Positioned at the top of the health care occupations' hierarchy [1], the medical profession has as its main goal the maintenance of its monopoly over general health policy, control over its work and the number in its profession, and the ability to set its own fees [68]. In this position of power, the medical profession's interests are "served by the existing social, economic and political structures" [[64] p. 2126] and thus the medical profession benefits from the maintenance of the *status quo*. This, according to Duckett's [68] application of Alford's structural interest perspective to the Australian health care system, is essentially the principal hallmark of the dominant interest, the professional monopolists. Thus, having achieved success in controlling the overall management of the childbirth process, it is in the medical profession's interest to ensure that its control is not eroded. In fact, the withdrawal of insurance cover for self-employed midwives has vindicated the medical profession's view that a midwife practicing autonomously, but more significantly, in the absence of any medical supervision, poses a high risk.

Even though the data reveal that the number of planned home births in Australia is small, the preference demonstrated by some women to birth at home, employing a midwife to provide care throughout their pregnancies, assist in the delivery process and provide care postnatally, suggests that in Australia the evolution of medicine's control over midwifery remains incomplete. It stands to reason then that 'competitors' in this 'industry' would welcome any opportunity that could improve or protect their market share, or suppress or eliminate competition – even if that competition is unlikely to pose a significant economic threat. The inability of self-employed midwives to secure professional indemnity cover has provided their rivals with such an opportunity.

As a professional group, midwives are entitled to be considered among Alford's professional monopolists. However, unlike the medical profession, midwives are not considered among the policy elites and their inclusion in this structural interests group is problematic. Unlike the medical profession, midwives have not had the benefit of other institutions defending their interests [68]. Instead, they have had to find their own political voice and argue for recognition as independent professionals and the legitimacy of their role in the childbirth continuum. The case study has revealed that the political voice of the midwifery profession was strengthened with the assistance of their supporters – largely women and their families who have formed the client base of self-employed midwives. Thus, from a structural interests perspective, such an alliance would lend the midwifery profession to be more appropriately positioned within the group of interests identified by Alford as the equal health advocates (or the community interest) [69].

Equal health advocates, according to Palmer and Short [[1] p. 44] "have in common their desire to improve the health care available in the community". However, while Palmer and Short suggest that equal health advocates have over time organised themselves into more effective lobby groups, and in the case of midwifery these include the Maternity Coalition and ACMI, compared with the professional monopolists equal health advocates are "relatively diffuse, not well organized, poorly financed, and generally lacking in bargaining power in the political arena" [[1] p. 44]. The nature of the debate waged in the public arena was, in part, demonstrable of this.

#### Media influence and the public debate

The media provides an important source of information for the public. However, the media's power lies not just in its ability to disseminate information but to determine the issues that are newsworthy and is therefore able to influence both public opinion and political decision making [58]. For those who are skilled at capturing its attention and harnessing that power, the media provides a vital conduit for shaping the policy agenda.

Over the period 2001 to 2003, the print media in Australia consistently published accounts that spiralling medical indemnity premiums and the threat of legal action were forcing the premature retirement of obstetricians as well as practitioners of other medical specialties [70]. In addition to the potential loss of services due to the early retirement of medical practitioners [71,72], the print media regularly reported on the profession's threats to withdraw services unless governments intervened to stem premium increases and address the problems of the medical indemnity insurance industry. These stories and those reporting the Australian Medical Association's claims that, if the medical indemnity situation remained unchanged "there will be no one left to look after mothers and babies" [73], unnerved the public and underpinned the push for political action.

Articles published in Australia's major newspapers over the period January 2001 to December 2005 informed the Australian public that insurers such as Guild Insurance and St Paul Insurance had decided to withdraw professional indemnity cover for midwives. They also reported on inability of the midwifery profession to secure professional indemnity cover from alternative insurers. Those articles set the momentum for a subsequent series of reports about the impact of the loss of insurance cover: self-employed midwives who would no longer practice; women who would birth at home without any midwifery support. However, that momentum failed to generate widespread public alarm.

Significantly, the plight of the midwives was overshadowed by the threat of resignations from the medical profession because of the increases in the cost of medical indemnity insurance premiums, particularly for the high-risk specialties such as obstetrics. If self-employed midwives ceased practicing because of their inability to secure cover, their decisions would directly affect only a small proportion of the Australian community. The loss of medical services however had the potential to affect a far greater proportion of that community.

It is also possible that the very nature of the media reports about the loss of professional indemnity insurance cover for midwives, and the impact of that loss, polarised public opinion. The public debate, as reported in the print media, was largely focused on hospital births versus home births. Midwives' inability to secure professional indemnity insurance cover and governments' inequitable treatment of small business operators became lost in a sea of debate that was focused on women's 'rights' to choose their style of birth and practitioner [12], the perils of an increasingly interventionist hospital system where caesarean rates were rising well above recommended levels for developed countries [74]-[76], the morality of pressuring women to opt for natural childbirth [77], and whether home births are safer than hospital births [78]. Given that only a small proportion of the population choose to birth at home, the media's focus on the issue of home births probably did little to further the midwives' cause in the eyes of the broader public.

Compared with the midwifery profession, the medical profession was better able to use the media to promote its issues, generate public concern and demand political action. The media therefore served the medical profession well by operating as a part of those institutions that defend the interests of the medical profession.

#### Formation of symbiotic relationships between governments and the medical profession

Governments (including the public service) are included amongst Alford's corporate rationalists "whose interests are served by the promotion of greater efficiency, effectiveness and equity in the provision of health services" [[1] p. 43]. The interests of the corporate rationalists and those of the professional monopolists are, from Alford's perspective, in competition [69]. Lewis [[64] p. 2126] suggests "corporate rationalists challenge the professional monopoly, by emphasizing rational planning and efficiency ahead of deference to the expertise of medical professionals". However, Australia's insurance 'crisis' and more specifically, events surrounding the inability of self-employed midwives to secure professional indemnity insurance cover, has revealed that an important symbiotic relationship between two usually combative interests was formed.

In the interest of political stability, and the effective and equitable performance of the health system, governments rely on the medical profession to continue to deliver services. In return, governments act to ensure that the interests of the medical profession are protected and that the *status quo *is maintained. The Australian Government's decisive and financially generous response to the demands of the medical profession during the medical indemnity 'crisis' is demonstrative of this relationship. So too is the Australian Government's steadfast inaction with respect to assisting self-employed midwives. This is illustrated by its unwillingness to invite midwifery representatives to the medical indemnity forum, its disregard for the National Maternity Action Plan and its refusal to recognize the insurance problems faced by self-employed midwives as a case of market failure.

Although from an economic perspective the potential threat to the medical profession posed by self-employed midwives could be considered negligible, the threat to the medical profession's dominance in the management of childbirth is potentially significant. Hence, by refraining from stepping in as an insurer of last resort, the Australian Government has ensured that the independent, small business status once held by self-employed midwives would not be reinstated. Without that status, any challenge to medicine's dominance posed by self-employed midwives, at least in the provision of private sector services, would be suppressed.

The symbiotic relationship between the medical profession and government has also extended to the governments of the states and territories. However, because of the reduced influence of the medical profession's union and associations at the state and territory level compared with their influence at the national level [64], and the less direct economic relationship between the medical profession and the states and territories, that relationship is less explicit.

#### Protection of market interests

The primary concern for states and territories, given their responsibility for the funding and conduct of public hospitals, is to position these hospitals so that they are protected from potential workforce shortages and any threats to the delivery of public sector programs. Arguably, this concern underpinned the various solutions devised by state and territory governments to support the continued provision of midwifery services in their jurisdictions. Further, it could be suggested that many of those solutions were designed, and their introduction timed, to take full advantage of the inability of self-employed midwives to secure professional indemnity insurance cover and their subsequent search for employment. For instance, ensuring the continuity of services was the principal concern for the West Australian Government's extension of insurance cover to those midwives engaged in the Fremantle Community Midwifery Program. The Government's support for the businesses of those midwives involved with that program was a secondary consideration. Similarly, the Victorian plan to give midwives greater professional autonomy in public hospitals was fundamentally designed to attract midwives into the public hospital system as well as to retain experienced employees.

The actions taken by the states and territories have served to maintain the *status quo *for the medical profession. Those midwives who wish to continue to use their skills but do not wish to practice uninsured were forced to seek employment within hospitals – settings in which the overall management of the childbirth rests with the medical profession. Further, even though the Victorian proposal seeks to give greater autonomy to midwives in public hospitals, it does not support midwives in private practice by, for example, granting private practice admitting rights into public hospitals. In this regard, the medical profession relies upon the state and territory governments to not further empower midwives by expanding their autonomy in the public hospital system and to refrain from stepping in as insurers of last resort for self-employed midwives which, in turn, would reinstate the independent, small business status once held by self-employed midwives.

### Does a 'bottom up' perspective provide any additional explanations for the reactions of Australian governments?

Earlier it was suggested that the protection of public sector interests underpinned the various solutions devised by state and territory governments to ensure the continued delivery of midwifery services in their respective jurisdictions. Of interest is whether those decisions, both in terms of their nature and scope, could have been influenced from the 'bottom up'.

Meier and Bohte [79] provide a useful standpoint from which such an enquiry can be launched. They contend that "bureaucracies are political institutions that are capable of representing the interests of citizens just as legislatures or executives do" [[79] p. 455]. Commentators such as Scott [80] and Sowa and Selden [81] temper this view by suggesting that such representation can only occur in bureaucracies with organisational structures and cultures that enable bureaucrats to exercise discretion. Within such an environment, the level of discretion able to be exercised by bureaucrats is dependent upon factors such as the degree of supervision exercised over employees, and the nature of the decisions being made [79]. Also significant are the values and attitudes held by the bureaucrat, and how he or she reads cues from the work environment in terms of the role that could be adopted towards representing outside interests [81].

For some bureaucrats their role may extend to acting as a 'trustee of minority interests' whereby he or she will assume the responsibility for "making a difference in policy outcomes for minorities, ensuring that their interests are served, and ensuring that they are given increasing access to the policy process" [[81] p.704]. Herbert, according to Sowa and Selden [[81] p. 704], suggests "minority communities often seek public administrators who will listen to them, who can communicate with them, who care about them". The likelihood of minority groups finding such sympathetic bureaucrats would be greater than those groups being able to find a receptive, higher-level decision-maker such as government minister. Further, it is within the bureaucracy that minority groups will find bureaucrats that have developed particular areas of expertise and are therefore well positioned to undertake research, develop options, draft documents and advise senior management and ministers on proposals and strategies. Bureaucrats from this group, because of their knowledge, are often appointed to committees, which, if external to their departments, provide valuable opportunities for broad networking and opinion sharing.

## Conclusion

The most significant issue arising from the story of the insurance problems besetting self-employed midwives is the apparent unwillingness of Australian governments to consider the withdrawal of the insurance market from writing professional indemnity insurance cover for self-employed midwives, and the impact on their businesses, as a case of genuine market failure that warranted some form of government intervention. Specifically, the form of intervention not forthcoming from governments was either the provision of government-based insurance cover in the absence of any insurance provider willing to extend professional indemnity to these small business operators or, when insurance providers were subsequently identified, the provision of financial assistance needed by self-employed midwives to secure such cover in the market place.

This article has suggested that there has been a general reluctance on the part of all Australian governments to protect the economic viability of the businesses of self-employed midwives following the insurance market's withdrawal of professional indemnity insurance cover. This same degree of reluctance was not evident when governments were called upon to assist the medical profession during its so called 'medical indemnity crisis' nor when premiums for public liability insurance reached levels that imperilled the viability of businesses or led to the cessation of community activities which in turn had the potential to result in the disruption and loss of community cohesion.

Consequently, this article has used a series of theoretical perspectives to explore why governments have reacted in the way that they have to the insurance problems that have beset self-employed midwives. These perspectives have revealed that the inequitable treatment of these small business operators, when compared with the support provided to the medical profession, stems from the capacity to influence governments' decision-making agendas.

Many factors impinge upon this capacity and these were explored from a 'top down' as well as a 'bottom up' perspective. A top down perspective revealed that to influence the agenda, self-employed midwives must overcome a seemingly insurmountable power imbalance between their profession and the medical profession. This article has suggested that the power of the latter is further enhanced by those institutional structures such as governments and the media who not only continue to serve the interests of the medical profession but who, in so doing, form important symbiotic relationships with the medical profession.

A 'bottom up' perspective has revealed that government bureaucracies are not passive subordinates that merely provide advice and implement decisions. Instead, it is suggested that government bureaucracies wield a high degree of influence over the policy decisions of governments. The fact that jurisdictions have rendered assistance to self-employed midwives (although this has effectively stopped short of reinstating self-employed midwives' independent status as small business operators) is demonstrative of the multidimensional nature of agenda setting and policymaking processes in state and territory bureaucracies. At the highest level, that is, the level of government ministers, the influence of the medical profession is strongest. Below this level is the bureaucracy and although its influence may not be as overt as that held by those in government or within the dominant interests, it is no less powerful and may be operating to undermine a policy agenda that is preventing solutions to the insurance problems of self-employed midwives being found.

Self-employed midwives failed to control the public debate and harness the power of the media to influence political action. The case study revealed that the public debate was largely driven by ideology and the media's attention was captured by those advocating home birthing as a superior choice to birthing in a medicalised hospital environment. Such a debate was never going to succeed in engendering widespread public support as only a very small proportion of the Australian population seeks the services of self-employed midwives, and the impact of the loss of these midwifery services would be minimal for the broader population. In comparison, the medical profession was masterful in using the media to engender public support – essentially by using the threat of service losses to generate a campaign of public fear and thus political action.

The debate did not focus on economic arguments and the amelioration of the perception of the high level of risk posed by self-employed midwives. Further, the campaign waged by self-employed midwives in both the media and with governments was not one that demanded that the services provided by self-employed midwives be accepted as a legitimate economic activity within the health sector and thus worthy of treatment like any other small business operators seeking to protect their livelihood. Once it became apparent that their initial demand for governments to provide professional indemnity cover would not be realised, self-employed midwives did not demand that the Australian Government either:

(i) require existing medical indemnity insurers to pool comparable risks and thus extend professional indemnity insurance cover to self-employed midwives (this could have been effected through existing regulatory arrangements); or

(ii) take action, as it had done on at least one other occasion, because clearly the market was failing to deliver "the 'socially optimal' amount of a product or a tolerable substitute" [[37] p. 10].

## Competing interests

The author declares that she has no competing interests.
